# Supplementary material for: A Plant-Based Food Guide Adapted for Low-Fat Diets: The VegPlate Low-Fat (VP_LF)
Source: Foods. 2024 Dec 15;13(24):4050. doi: 10.3390/foods13244050 (PMC11728239; doi:10.3390/foods13244050)
Supplement: Supplementary file 1 [file foods-13-04050-s001.zip › foods-3345250-supplementary.pdf]

Table S1. Number of servings suggested for calorie intakes from 3100 to 4000 kcal.

	Grains	Protein-rich foods	Vegetables	Fruits	n-3-rich foods	Discretionary calories
3100	16	4	10	9	2	350
3200	16	4	10	10	2	381
3300	17	4	11	10	2	378
3400	17	4	11	10	2	478
3500	18	4	12	10	2	474
3600	18	4	12	10	2	574
3700	19	4	13	10	2	571
3800	19	4	13	10	2	671
3900	20	4	14	10	2	668
4000	20	4	14	10	2	768

Table S2. Sample 2000 kcal vegan low-fat menu.

	Grains (G)	Protein-rich foods (P)	Vegetables (V)	Fruits (F)	n-3-rich foods (n3)
2000 kcal	10	4	8	4	1
Breakfast	2	1	-	1	0.5
Morning snack	-	-	2	-	-
Lunch	4	2	3	-	-
Afternoon snack	-	-	-	2	-
Dinner	4	1	3	-	0.5
Evening snack	-	-	-	1	-
**Ingredients**					
Breakfast	Whole grain bread 60 g (2G)	Soymilk 200 g (1P)	-	Banana 150 g (1F)	Walnuts cream 15 g (0.5 n3)
Morning snack	--	-	Carrots 70 g, celery 70 g and green radicchio 60 g (tot 200 g) (2V)	-	-
Lunch	Whole grain pasta 60 g Whole grain bread 60 g (4G)	Beans 60 g (2P)	Broccoli 180 g, onion 20 g, red radicchio 50 g, rocket 50 g (tot 300 g) (3V) Thymus 5 g	-	-
Afternoon snack	-	-	-	Kiwifruits, orange 300 g (2F)	-
Dinner	Spelt 60 g Whole grain bread 60 g (4G)	Tofu 80 g (1P)	Porcini 180 g, onions 20 g, chicory 100 g (tot 300 g) (3V) Rosemary 5 g Lemon juice 5 g	-	Flaxseed oil 3 g (0.5 n3)
Evening snack	-	-	-	Persimmon 150 g (1F)	
**Sample menu***					
Breakfast	Whole grain bread with walnut cream and sliced banana; soymilk.				
Morning snack	Vegetable juice with carrots, celery and green radicchio.				
Lunch	Whole grain pasta with broccoli, onions and thymus; mixed salad with beans, red radicchio and rocket; whole grain bread.				
Afternoon snack	Kiwifruits and orange.				
Dinner	Spelt with onions and porcini; tofu with chicory, rosemary, lemon juice and flaxseed oil; whole grain bread.				
Evening snack	Persimmon.				
**Nutrient composition**					
Total Energy (kcal)	1749				
Discretionary Calories (kcal)	251				

Discretionary Calories (%totalEn)	12.6%
Protein (g)	85.16
Fats (g)	30.5
Fats (%totalEn)	13.8%
Calcium** (mg)	1191
Iron (mg)	37.5
Zinc (mg)	12.6
Vitamin B_1 (mg)	2.14
Vitamin B_2 (mg)	2.68
Vitamin B_3 (mg)	31.41

*The different foods can be redistributed according to personal choices

**Without calcium from water
